# Supplementary material for: Analysis of Photosynthetic Characteristics and Screening High Light-Efficiency Germplasm in Sugarcane
Source: Plants (Basel). 2024 Feb 22;13(5):587. doi: 10.3390/plants13050587 (PMC10935250; doi:10.3390/plants13050587)
Supplement: Supplementary file 1 [file plants-13-00587-s001.zip › Table S1.pdf]

Table S1. Information on 258 sugarcane genotype parentages

NO.	Genotype	Parental combination	NO.	Genotype	Parental combination	NO.	Genotype	Parental combination
1	3203	Unknown	87	14-21107	FN02-3924×YR09-74	173	16-1335	ROC25×YZ89-7
2	3717	ROC25×YZ89-7	88	15-11106	FN02-6404×HoCP00-1142	174	16-1342	ROC25×YZ89-7
3	6010	Unknown	89	15-14701	YT00-319×GT00-122	175	16-136	ROC25×YZ89-7
4	6101	CT89-103×ROC22	90	15-16850	CP89-2143×YT00-236	176	16-137	ROC25×YZ89-7
5	6105	Unknown	91	15-22809	FN02-3924×YC04-55	177	16-142	HoCP01-564×YC06-92
6	8914	Unknown	92	15-23304	Unknown	178	16-144	HoCP01-564×YC06-92
7	10-228	Unknown	93	16-10002	YT00-236×YC06-61	179	16-151	CP08-2506×CP05-1616
8	11-601	Unknown	94	16-11203	YT85-177×GT96-211	180	16-154	CP08-2506×CP05-1616
9	12-106	Unknown	95	16-11708	YT92-1287×CP94-1100	181	16-1612	CP94-2059×CP97-1777
10	14-002	Unknown	96	16-11905	YT93-159×ROC22	182	16-163	CP94-2059×CP97-1777
11	14-509	Unknown	97	16-12026	YT93-159×GT03-8	183	16-167	CP94-2059×CP97-1777
12	16-401	Unknown	98	16-12506	YT93-159×YT91-976	184	16-168	CP94-2059×CP97-1777
13	16-615	Unknown	99	16-12509	YT93-159×YT91-976	185	16-1811	CPCL05-1201×CP05-1616
14	16-803	Unknown	100	16-12512	YT93-159×YT91-976	186	16-182	CPCL05-1201×CP05-1616
15	19-607	Unknown	101	16-15220	GT00-122×ROC22	187	16-184	CPCL05-1201×CP05-1616
16	20-718	Unknown	102	16-22402	Unknown	188	16-186	CPCL05-1201×CP05-1616
17	24201	Unknown	103	16-22419	Unknown	189	16-187	CPCL05-1201×CP05-1616
18	35365	YT93-159×YC84-153	104	06-0918	Unknown	190	16-188	CPCL05-1201×CP05-1616
19	40375	YT00-236×GT94-38	105	09-175	Unknown	191	16-192	CP08-2506×CP08-1553
20	11-2819	FN94-0403×YR05-679	106	11-11319	FN02-3924×YR05-326	192	16-195	CP08-2506×CP08-1553
21	12-1801	FN94-0403×ROC22	107	12-14602	MT96-6016×YZ89-7	193	16-198	CP08-2506×CP08-1553
22	12-6403	YZ03-194×FN94-0403	108	12-17204	YT99-66×ROC22	194	16-222	B35-9×CP08-1553
23	13-1105	YC06-91×YZ02-588	109	12-34	Co1001×ROC22	195	16-223	B35-9×CP08-1553
24	13-4007	YC07-71×FN02-6427	110	13-11008	GT96-211×FN02-6427	196	16-224	B35-9×CP08-1553
25	14-2149	FN95-1702×GZ74-141	111	13-11919	LC04-13×GT00-122	197	16-225	B35-9×CP08-1553

26	14-2244	GT00-122×YT89-113	112	13-14812	ROC22×YT93-124	198	16-226	B35-9×CP08-1553
27	14-2720	GT89-5×ROC22	113	14-12012	ROC25×LC04-256	199	16-231	CPCL05-1201×CP97-1777
28	14-2802	GT92-66×ROC22	114	14-1854	FN94-0744×LC03-182	200	16-232	CPCL05-1201×CP97-1777
29	14-3508	LC05-292×YT01-72	115	14-4315	CP81-1254×ROC22	201	16-251	CPCL05-1201×CP97-1777
30	14-3902	Neijiang86-117×YT91-976	116	14-434	CP81-1254×ROC22	202	16-253	CPCL05-1201×CP97-1777
31	14-5603	YT93-159×ROC22	117	14-8705	Pma98-40×YR05-704	203	16-255	CPCL05-1201×CP97-1777
32	14-8004	GZ90-76×ROC22	118	15-18106	ROC25×FN91-4621	204	16-256	CPCL05-1201×CP97-1777
33	14-8009	GZ90-76×ROC22	119	15-42	Qiantang4×YR10-636	205	16-262	CP08-2506×CP84-1198
34	14-8704	Pma98-40×YR05-704	120	15-421	Qiantang4×YR10-636	206	16-264	CP08-2506×CP84-1198
35	14-8903	Yunkai03-206×YR05-704	121	15-451	YR10-527×YZ05-49	207	16-271	CP16-0916×CP16-0916
36	15-0102	CP81-1254×FN02-6427	122	15-452	YR10-527×YZ05-49	208	16-453	Unknown
37	15-1005	DZ93-88×Neijiang03-218	123	15-453	YR10-527×YZ05-49	209	16-831	Unknown
38	15-1103	FN02-3924×YC06-61	124	15-4818	YC07-71×GT00-122	210	16-832	Unknown
39	15-1106	FN02-3924×YC06-61	125	15-701	DZ99-36×YR10-498	211	CP01-1372	Unknown
40	15-1743	FN95-1702×YT99-66	126	15-702	DZ99-36×YR10-498	212	FG2	ROC10×YC84-153
41	15-2007	GT02-761×Neijiang03-218	127	15-791	DZ09-78×YR10-498	213	FG3	Q61×CP49-50
42	15-2010	GT02-761×Neijiang03-218	128	15-793	DZ09-78×YR10-498	214	FN0335	GT 00-122×ROC10
43	15-3303	GT94-119×GT00-122	129	15-794	DZ09-78×YR10-498	215	FN04-3504	ROC25×CP84-1198
44	15-4203	Neijiang03-218×GT83-492	130	15-9904	GT94-119×DZ03-83	216	FN10-0574	CP72-1210×YZ94-343
45	15-4513	YC07-65×GT00-122	131	15-W3	CP94-2059×CP97-1777	217	Fujiandaye	Unknown
46	15-5306	YT00-236×ROC22	132	16-041	YT94-128×ROC25	218	Ganjiang18	ROC1×YC71-374
47	15-5404	YT00-236×GT00-122	133	16-043	YT94-128×ROC25	219	GT02-390	YT85-177×GT92-66
48	15-6008	YT03-393×YT01-71	134	16-063	YT94-128×YC07-71	220	GT03-351	Unknown
49	15-6201	YT91-976×HoCP95-988	135	16-064	YT94-128×YC07-71	221	GT42	ROC22×GT92-66
50	15-6204	YT91-976×HoCP95-988	136	16-065	YT94-128×YC07-71	222	GT05-378	GT93-102×ROC22
51	15-6402	YT91-976×YZ02-2540	137	16-066	YT94-128×YC07-71	223	GT92-66	YT83-257×YC71-374
52	16-0628	YT00-236×YT99-66	138	16-0812	YT94-128×ROC22	224	GT94-119	Ganzhe76-65×YC71-374

53	16-1015	YT91-976×CP84-1198	139	16-084	YT94-128×ROC22	225	GUC10	Unknown
54	16-1715	CP94-1100×GT03-3089	140	16-087	YT94-128×ROC22	226	GUC13	CP00-1100×Q209
55	16-2026	HoCP00-2218×YC07-71	141	16-088	YT94-128×ROC22	227	GUC16	HoCP01-157×CP14-096
56	16-3205	ROC22×GT00-122	142	16-091	CT89-103×ROC22	228	GUC17	Unknown
57	16-3417	ROC28×CP94-1100	143	16-0911	CT89-103×ROC22	229	GUC2	CP88-1762×CP96-1252
58	16-5402	FN0713×YC07-71	144	16-0913	CT89-103×ROC22	230	GUC21	Unknown
59	16-7010	GT03-8×GT02-761	145	16-0914	CT89-103×ROC22	231	GUC23	HoCP01-157×CP14-096
60	16-7019	GT03-8×GT02-761	146	16-0916	CT89-103×ROC22	232	GUC25	CP89-2143×CP72-1210
61	16-7506	GT92-66×FN02-6427	147	16-0917	CT89-103×ROC22	233	GUC29	Unknown
62	16-7705	GT96-143×YC07-71	148	16-092	CT89-103×ROC22	234	GUC3	CP88-1762×CP96-1252
63	16-7719	GT96-143×YC07-71	149	16-0920	CT89-103×ROC22	235	GUC31	Unknown
64	16-7722	GT96-143×YC07-71	150	16-0924	CT89-103×ROC22	236	GUC35	CP89-2143×CP72-1210
65	16-8701	Neijiang97-128×CP94-1100	151	16-0926	CT89-103×ROC22	237	GUC41	Unknown
66	16-8716	Neijiang97-128×CP94-1100	152	16-0927	CT89-103×ROC22	238	GUC7	Unknown
67	16-8801	Neijiang97-128×HoCP05-902	153	16-0928	CT89-103×ROC22	239	GUC8	HoCP01-157×CP14-096
68	16-8804	Neijiang97-128×HoCP05-902	154	16-0929	CT89-103×ROC22	240	LC05-129	Unknown
69	11-20318	YT01-127×GT94-119	155	16-093	CT89-103×ROC22	241	ROC16	F171×74-575
70	12-12803	GT94-119×YN73-204	156	16-0930	CT89-103×ROC22	242	ROC22	ROC5×69-463
71	12-20318	CP94-1100×YR05-367	157	16-0931	CT89-103×ROC22	243	ROC27	F176×CP58-48
72	13-18402	YT01-23×GT00-122	158	16-0934	CT89-103×ROC22	244	Shuidian25	Unknown
73	13-21501	Unknown	159	16-0936	CT89-103×ROC22	245	Taiyin14	Unknown
74	14-10006	YR09-28×YZ03-422	160	16-0939	CT89-103×ROC22	246	TB1	FN90-4304 GMOs
75	14-12506	ROC25×YT92-1287	161	16-0941	CT89-103×ROC22	247	TB11	FN90-4304 GMOs
76	14-12712	ROC28×HoCP00-1142	162	16-0942	CT89-103×ROC22	248	TB3	FN90-4304 GMOs
77	14-14325	GT02-901×ROC23	163	16-0953	CT89-103×ROC22	249	X	Unknown
78	14-14707	GT05-3084×ROC22	164	16-0954	CT89-103×ROC22	250	Xi096	Unknown

79	14-15220	LC03-1137×GT00-122	165	16-096	CT89-103×ROC22	251	YC64-389	Co419×YC62-70
80	14-15239	LC03-1137×GT00-122	166	16-098	CT89-103×ROC22	252	YG24	YT92-1287×YT93-159
81	14-15418	LC04-13×CP94-1100	167	16-104	ROC20×GT89-5	253	YG39	YT94-128×YT93-159
82	14-18504	YT94-128×ROC20	168	16-106	ROC20×GT89-5	254	YR03-425	ROC10×YR99-546
83	14-18509	YT94-128×ROC20	169	16-1322	ROC25×YZ89-7	255	YR99-596	Co419×YC85-881
84	14-19220	CP80-1827×ROC24	170	16-1329	ROC25×YZ89-7	256	GZ74-141	Huanan56-12×YC58-47
85	14-20701	Q208×YZ00-45	171	16-1330	ROC25×YZ89-7	257	GZ96-126	YT85-177×ROC10
86	14-21001	YG60×YR10-291	172	16-1331	ROC25×YZ89-7	258	ZZ9	YZ89-7×ROC25
