# Supplementary material for: Analysis of Photosynthetic Characteristics and Screening High Light-Efficiency Germplasm in Sugarcane
Source: Plants (Basel). 2024 Feb 22;13(5):587. doi: 10.3390/plants13050587 (PMC10935250; doi:10.3390/plants13050587)
Supplement: Supplementary file 1 [file plants-13-00587-s001.zip › Table S2.pdf]

Table S2. Cluster and differential analysis of photosynthetic efficiency of sugarcane genotypes.

NO.	Genotype	Fm	Fo	Fv/Fm	Fv/Fo	Fv	Y(NO)	SPAD	Clustering	Discriminant	Posterior probability
1	6105	1.051	0.241	0.769	3.387	0.809	0.231	48.185	HPE	HPE	0.840
2	24201	0.983	0.221	0.774	3.435	0.761	0.226	44.033	HPE	HPE	0.638
3	09-175	1.061	0.237	0.776	3.493	0.824	0.224	45.463	HPE	HPE	0.812
4	11-11319	1.030	0.238	0.769	3.340	0.792	0.231	48.137	HPE	HPE	0.739
5	12-20318	1.029	0.237	0.769	3.362	0.790	0.231	45.826	HPE	HPE	0.516
6	12-6403	1.024	0.234	0.771	3.389	0.789	0.229	46.822	HPE	HPE	0.788
7	14-10006	1.099	0.252	0.770	3.381	0.847	0.230	47.863	HPE	HPE	0.772
8	14-12012	1.053	0.242	0.771	3.394	0.811	0.229	48.015	HPE	HPE	0.859
9	14-12506	1.03	0.234	0.772	3.405	0.796	0.228	46.915	HPE	HPE	0.840
10	14-14707	0.907	0.192	0.788	3.717	0.715	0.212	48.778	HPE	HPE	1.000
11	14-15239	0.884	0.197	0.776	3.484	0.686	0.224	46.630	HPE	HPE	0.754
12	14-15418	1.065	0.249	0.766	3.294	0.816	0.234	49.000	HPE	HPE	0.621
13	14-18504	1.069	0.245	0.770	3.379	0.823	0.230	47.033	HPE	HPE	0.693
14	14-21001	0.940	0.212	0.774	3.441	0.728	0.226	46.052	HPE	HPE	0.797
15	14-2244	0.947	0.207	0.780	3.545	0.739	0.220	42.270	HPE	HPE	0.791
16	14-2802	1.076	0.248	0.770	3.379	0.828	0.230	47.600	HPE	HPE	0.765
17	14-3508	0.978	0.218	0.774	3.459	0.760	0.226	45.096	HPE	HPE	0.810
18	14-8004	1.001	0.219	0.785	3.664	0.786	0.215	48.541	HPE	HPE	0.999
19	14-8704	1.014	0.229	0.775	3.460	0.786	0.225	48.763	HPE	HPE	0.978
20	15-0102	1.136	0.260	0.772	3.406	0.876	0.228	48.059	HPE	HPE	0.797
21	15-1103	0.964	0.219	0.773	3.437	0.745	0.227	49.800	HPE	HPE	0.919
22	15-11106	1.015	0.222	0.781	3.582	0.793	0.219	45.815	HPE	HPE	0.975
23	15-16850	1.057	0.228	0.784	3.642	0.829	0.216	46.189	HPE	HPE	0.986
24	15-18106	1.125	0.252	0.776	3.471	0.873	0.224	46.552	HPE	HPE	0.802
25	15-22809	0.916	0.201	0.782	3.589	0.716	0.218	44.478	HPE	HPE	0.975
26	15-23304	1.001	0.225	0.774	3.450	0.775	0.226	43.333	HPE	HPE	0.516
27	15-3303	1.094	0.247	0.774	3.431	0.845	0.226	49.196	HPE	HPE	0.958
28	15-42	1.103	0.250	0.776	3.488	0.857	0.224	46.219	HPE	HPE	0.816
29	15-4203	1.064	0.229	0.783	3.627	0.833	0.217	46.096	HPE	HPE	0.980
30	15-451	1.012	0.230	0.772	3.408	0.780	0.228	48.415	HPE	HPE	0.915
31	15-452	1.045	0.232	0.778	3.511	0.813	0.222	46.296	HPE	HPE	0.939
32	15-4818	0.971	0.220	0.772	3.419	0.751	0.228	43.881	HPE	HPE	0.513
33	15-701	1.114	0.239	0.784	3.644	0.874	0.216	45.159	HPE	HPE	0.946
34	15-793	0.957	0.214	0.775	3.498	0.744	0.225	46.019	HPE	HPE	0.908
35	15-W3	0.879	0.197	0.778	3.527	0.685	0.222	38.581	HPE	MPE**	0.681
36	16-041	1.022	0.225	0.779	3.538	0.797	0.221	51.504	HPE	HPE	0.999
37	16-0628	1.018	0.219	0.784	3.654	0.799	0.216	40.878	HPE	HPE	0.674
38	16-063	0.954	0.211	0.775	3.476	0.740	0.225	42.363	HPE	HPE	0.511

39	16-0812	0.960	0.215	0.774	3.443	0.743	0.226	51.237	HPE	HPE	0.944
40	16-084	1.010	0.224	0.776	3.482	0.785	0.224	51.870	HPE	HPE	0.996
41	16-0916	1.001	0.230	0.778	3.515	0.779	0.222	48.944	HPE	HPE	0.992
42	16-0920	0.889	0.200	0.775	3.455	0.690	0.225	45.619	HPE	HPE	0.588
43	16-0924	1.059	0.246	0.767	3.330	0.812	0.233	47.407	HPE	HPE	0.545
44	16-0930	0.943	0.211	0.778	3.508	0.733	0.222	43.285	HPE	HPE	0.801
45	16-0934	1.069	0.241	0.779	3.523	0.832	0.221	47.970	HPE	HPE	0.978
46	16-0941	1.060	0.237	0.775	3.485	0.823	0.225	45.541	HPE	HPE	0.796
47	16-098	1.026	0.238	0.768	3.337	0.789	0.232	48.837	HPE	HPE	0.722
48	16-1015	0.977	0.215	0.781	3.571	0.763	0.219	45.207	HPE	HPE	0.969
49	16-104	1.006	0.223	0.777	3.501	0.782	0.223	45.737	HPE	HPE	0.932
50	16-106	0.915	0.203	0.779	3.549	0.712	0.221	41.100	HPE	HPE	0.668
51	16-11708	1.059	0.235	0.778	3.501	0.823	0.222	44.448	HPE	HPE	0.742
52	16-12026	0.974	0.218	0.775	3.469	0.755	0.225	47.126	HPE	HPE	0.946
53	16-12509	1.092	0.232	0.787	3.701	0.859	0.213	43.159	HPE	HPE	0.911
54	16-1322	1.036	0.241	0.768	3.315	0.795	0.232	47.530	HPE	HPE	0.553
55	16-1342	1.067	0.233	0.78	3.572	0.833	0.22	44.811	HPE	HPE	0.891
56	16-137	0.950	0.214	0.773	3.436	0.736	0.227	48.707	HPE	HPE	0.843
57	16-151	0.954	0.213	0.778	3.527	0.742	0.222	46.893	HPE	HPE	0.976
58	16-154	1.035	0.238	0.769	3.336	0.797	0.231	47.744	HPE	HPE	0.685
59	16-1811	1.059	0.243	0.770	3.377	0.816	0.230	47.448	HPE	HPE	0.777
60	16-184	1.031	0.236	0.769	3.341	0.794	0.231	47.056	HPE	HPE	0.627
61	16-192	1.098	0.231	0.788	3.741	0.865	0.212	46.367	HPE	HPE	0.994
62	16-195	0.983	0.223	0.772	3.392	0.759	0.228	44.078	HPE	HPE	0.463
63	16-2026	0.989	0.214	0.785	3.647	0.776	0.215	44.115	HPE	HPE	0.973
64	16-226	1.050	0.247	0.767	3.326	0.804	0.233	49.719	HPE	HPE	0.752
65	16-231	0.967	0.220	0.772	3.410	0.747	0.228	47.185	HPE	HPE	0.746
66	16-253	0.911	0.200	0.781	3.563	0.711	0.219	45.652	HPE	HPE	0.977
67	16-255	0.994	0.224	0.775	3.450	0.77	0.226	47.822	HPE	HPE	0.951
68	16-262	0.981	0.210	0.787	3.709	0.771	0.213	41.352	HPE	HPE	0.908
69	16-271	0.928	0.207	0.776	3.498	0.721	0.224	46.341	HPE	HPE	0.919
70	16-401	0.921	0.208	0.774	3.443	0.713	0.226	43.393	HPE	HPE	0.552
71	16-7010	1.010	0.220	0.781	3.575	0.789	0.219	46.863	HPE	HPE	0.988
72	16-7506	0.912	0.200	0.783	3.612	0.713	0.217	47.152	HPE	HPE	0.996
73	16-8716	0.882	0.199	0.778	3.512	0.686	0.222	45.100	HPE	HPE	0.843
74	19-607	0.956	0.213	0.777	3.513	0.742	0.223	46.356	HPE	HPE	0.957
75	FG2	0.975	0.209	0.785	3.650	0.765	0.215	46.330	HPE	HPE	0.996
76	FN0335	0.927	0.204	0.781	3.577	0.724	0.219	45.874	HPE	HPE	0.986
77	FN10-0574	0.991	0.222	0.774	3.476	0.769	0.226	47.141	HPE	HPE	0.938
78	GT03-351	0.999	0.230	0.769	3.349	0.769	0.231	47.407	HPE	HPE	0.604
79	GT42	1.004	0.231	0.771	3.39	0.774	0.229	46.596	HPE	HPE	0.741
80	GT05-378	1.102	0.244	0.778	3.511	0.857	0.222	44.456	HPE	HPE	0.641

81	GUC13	0.958	0.218	0.776	3.463	0.743	0.224	44.615	HPE	HPE	0.806
82	GUC23	0.929	0.210	0.776	3.487	0.719	0.224	42.385	HPE	HPE	0.577
83	GZ74-141	1.060	0.247	0.767	3.328	0.814	0.233	47.748	HPE	HPE	0.627
84	ROC22	1.009	0.227	0.773	3.444	0.781	0.227	48.100	HPE	HPE	0.944
85	X	1.030	0.226	0.780	3.561	0.803	0.220	47.141	HPE	HPE	0.985
86	YG39	1.060	0.236	0.777	3.509	0.824	0.223	45.815	HPE	HPE	0.887
87	3717	0.935	0.226	0.759	3.161	0.709	0.241	41.959	LPE	LPE	0.970
88	6101	0.925	0.213	0.768	3.336	0.712	0.232	45.367	LPE	LPE	0.865
89	8914	0.967	0.240	0.753	3.099	0.727	0.247	48.478	LPE	LPE	0.999
90	35365	0.997	0.244	0.754	3.066	0.751	0.246	42.533	LPE	LPE	0.948
91	40375	1.061	0.262	0.755	3.122	0.800	0.245	52.200	LPE	LPE	0.981
92	112819	1.138	0.286	0.749	3.008	0.851	0.251	46.419	LPE	LPE	0.615
93	1415220	0.978	0.237	0.757	3.125	0.741	0.243	44.896	LPE	LPE	0.983
94	06-0918	0.966	0.233	0.758	3.15	0.733	0.242	48.941	LPE	LPE	0.997
95	10-228	0.900	0.207	0.772	3.42	0.694	0.228	42.170	LPE	LPE	0.625
96	11-20318	1.031	0.246	0.759	3.164	0.784	0.241	46.167	LPE	LPE	0.880
97	11-601	0.930	0.212	0.770	3.366	0.717	0.230	46.211	LPE	LPE	0.720
98	12-106	0.970	0.235	0.758	3.195	0.735	0.242	46.074	LPE	LPE	0.986
99	12-12803	0.963	0.226	0.764	3.259	0.737	0.236	45.259	LPE	LPE	0.911
100	12-14602	0.900	0.207	0.770	3.366	0.693	0.230	46.296	LPE	LPE	0.834
101	12-17204	1.108	0.269	0.757	3.146	0.839	0.243	48.663	LPE	LPE	0.648
102	12-34	1.070	0.262	0.754	3.09	0.808	0.246	46.859	LPE	LPE	0.903
103	13-11008	0.981	0.230	0.764	3.243	0.750	0.236	44.300	LPE	LPE	0.816
104	13-11919	1.042	0.252	0.759	3.189	0.790	0.241	44.922	LPE	LPE	0.639
105	13-14812	0.901	0.215	0.76	3.184	0.686	0.240	48.596	LPE	LPE	0.999
106	13-18402	0.970	0.231	0.761	3.190	0.739	0.239	42.796	LPE	LPE	0.885
107	13-21501	1.076	0.268	0.749	3.022	0.808	0.251	44.019	LPE	LPE	0.832
108	14-002	0.984	0.228	0.765	3.299	0.756	0.235	48.326	LPE	LPE	0.809
109	14-12712	0.902	0.212	0.763	3.249	0.690	0.237	45.119	LPE	LPE	0.994
110	14-1854	1.032	0.249	0.758	3.145	0.782	0.242	42.711	LPE	LPE	0.576
111	14-2149	1.062	0.254	0.762	3.253	0.808	0.238	47.715	LPE	LPE	0.532
112	14-3902	0.913	0.215	0.762	3.226	0.698	0.238	41.619	LPE	LPE	0.961
113	14-434	0.978	0.233	0.760	3.203	0.745	0.240	42.226	LPE	LPE	0.790
114	14-509	1.053	0.258	0.754	3.092	0.795	0.246	43.967	LPE	LPE	0.759
115	14-5603	1.045	0.270	0.743	2.919	0.776	0.257	50.304	LPE	LPE	1.000
116	14-8009	1.033	0.246	0.761	3.238	0.787	0.239	43.837	LPE	MPE**	0.556
117	14-8705	0.922	0.212	0.770	3.380	0.710	0.230	45.981	LPE	LPE	0.727
118	14-8903	1.065	0.254	0.762	3.220	0.811	0.238	48.644	LPE	LPE	0.616
119	15-14701	0.985	0.228	0.766	3.307	0.757	0.234	48.189	LPE	LPE	0.747
120	15-1743	0.940	0.220	0.764	3.261	0.720	0.236	42.530	LPE	LPE	0.876
121	15-2010	0.951	0.224	0.763	3.246	0.727	0.237	40.848	LPE	LPE	0.714
122	15-421	0.996	0.235	0.767	3.308	0.764	0.233	47.974	LPE	LPE	0.609

123	15-453	1.039	0.248	0.759	3.192	0.791	0.241	46.852	LPE	LPE	0.862
124	15-5306	0.918	0.215	0.768	3.322	0.704	0.232	41.689	LPE	LPE	0.764
125	15-6008	0.985	0.228	0.767	3.317	0.757	0.233	47.085	LPE	LPE	0.619
126	15-6201	0.913	0.214	0.766	3.281	0.699	0.234	44.430	LPE	LPE	0.967
127	15-6402	0.930	0.231	0.753	3.095	0.699	0.247	48.163	LPE	LPE	1.000
128	15-702	0.965	0.225	0.766	3.290	0.740	0.234	43.548	LPE	LPE	0.706
129	15-9904	1.038	0.253	0.757	3.148	0.785	0.243	48.015	LPE	LPE	0.953
130	16-043	1.034	0.244	0.764	3.282	0.789	0.236	51.822	LPE	LPE	0.532
131	16-087	0.981	0.231	0.765	3.268	0.750	0.235	47.581	LPE	LPE	0.859
132	16-088	0.896	0.202	0.773	3.417	0.691	0.227	50.419	LPE	HPE**	0.559
133	16-091	0.911	0.226	0.749	3.019	0.684	0.251	44.926	LPE	LPE	1.000
134	16-0911	0.991	0.233	0.765	3.269	0.758	0.235	46.426	LPE	LPE	0.815
135	16-0913	0.985	0.232	0.765	3.297	0.754	0.235	46.044	LPE	LPE	0.792
136	16-0917	0.889	0.211	0.762	3.233	0.677	0.238	49.567	LPE	LPE	0.998
137	16-092	0.945	0.219	0.768	3.326	0.727	0.232	44.204	LPE	LPE	0.761
138	16-0926	0.901	0.214	0.762	3.242	0.686	0.238	45.059	LPE	LPE	0.995
139	16-0927	0.918	0.209	0.771	3.385	0.709	0.229	45.548	LPE	LPE	0.691
140	16-0929	0.967	0.236	0.755	3.112	0.731	0.245	44.996	LPE	LPE	0.994
141	16-093	0.947	0.219	0.768	3.359	0.728	0.232	45.922	LPE	LPE	0.740
142	16-0931	0.93	0.22	0.763	3.237	0.71	0.237	44.319	LPE	LPE	0.977
143	16-0942	1.045	0.252	0.756	3.132	0.792	0.244	45.122	LPE	LPE	0.836
144	16-096	0.981	0.236	0.758	3.167	0.745	0.242	46.370	LPE	LPE	0.985
145	16-10002	0.984	0.228	0.767	3.325	0.754	0.233	45.189	LPE	LPE	0.599
146	16-11203	0.826	0.188	0.771	3.373	0.637	0.229	42.041	LPE	LPE	0.979
147	16-11905	0.851	0.199	0.768	3.369	0.651	0.232	47.341	LPE	LPE	0.978
148	16-12512	1.085	0.263	0.755	3.118	0.821	0.245	49.452	LPE	LPE	0.934
149	16-1329	0.978	0.23	0.764	3.282	0.748	0.236	45.900	LPE	LPE	0.858
150	16-1331	0.898	0.207	0.768	3.337	0.691	0.232	46.563	LPE	LPE	0.945
151	16-1335	1.017	0.238	0.767	3.297	0.780	0.234	45.807	LPE	LPE	0.412
152	16-142	0.969	0.237	0.756	3.133	0.733	0.244	44.567	LPE	LPE	0.988
153	16-144	0.989	0.238	0.759	3.166	0.751	0.241	45.111	LPE	LPE	0.952
154	16-163	0.876	0.200	0.771	3.380	0.676	0.229	43.752	LPE	LPE	0.892
155	16-168	0.872	0.211	0.757	3.139	0.660	0.243	45.785	LPE	LPE	1.000
156	16-1715	1.114	0.261	0.763	3.259	0.853	0.237	51.541	LPE	HPE**	0.741
157	16-182	0.972	0.23	0.762	3.233	0.741	0.238	43.393	LPE	LPE	0.854
158	16-188	0.907	0.206	0.772	3.394	0.701	0.228	42.919	LPE	LPE	0.643
159	16-198	0.941	0.224	0.761	3.218	0.716	0.239	37.741	LPE	LPE	0.522
160	16-22419	0.916	0.214	0.766	3.298	0.702	0.234	43.381	LPE	LPE	0.938
161	16-225	0.869	0.196	0.774	3.429	0.673	0.226	43.437	LPE	LPE	0.722
162	16-232	0.949	0.230	0.756	3.124	0.718	0.244	44.504	LPE	LPE	0.995
163	16-251	0.98	0.229	0.765	3.276	0.750	0.235	48.056	LPE	LPE	0.845
164	16-264	0.862	0.204	0.76	3.217	0.657	0.240	44.244	LPE	LPE	0.999

165	16-3205	0.985	0.244	0.751	3.035	0.741	0.250	38.256	LPE	LPE	0.821
166	16-3417	0.979	0.232	0.762	3.226	0.747	0.238	41.737	LPE	LPE	0.641
167	16-5402	0.848	0.202	0.763	3.244	0.645	0.237	43.230	LPE	LPE	0.998
168	16-615	0.951	0.220	0.769	3.366	0.731	0.231	45.678	LPE	LPE	0.668
169	16-7719	0.981	0.228	0.767	3.306	0.753	0.233	43.311	LPE	MPE**	0.462
170	16-803	1.108	0.266	0.760	3.176	0.842	0.240	51.444	LPE	LPE	0.621
171	16-831	0.940	0.221	0.764	3.253	0.719	0.236	47.167	LPE	LPE	0.971
172	16-8701	0.914	0.212	0.767	3.304	0.702	0.233	41.422	LPE	LPE	0.840
173	16-8804	0.907	0.210	0.767	3.328	0.697	0.233	40.874	LPE	LPE	0.793
174	20-718	0.859	0.209	0.757	3.156	0.650	0.243	48.693	LPE	LPE	1.000
175	6010A	1.079	0.263	0.757	3.120	0.816	0.243	47.289	LPE	LPE	0.791
176	CP01-1372	0.938	0.225	0.759	3.145	0.711	0.241	40.711	LPE	LPE	0.943
177	FG3	0.862	0.197	0.769	3.376	0.664	0.231	39.074	LPE	LPE	0.834
178	FN04-3504	0.857	0.194	0.773	3.427	0.663	0.227	39.393	LPE	LPE	0.681
179	Fujiandaye	0.980	0.228	0.766	3.293	0.753	0.234	48.941	LPE	LPE	0.757
180	Ganjiang18	0.889	0.214	0.760	3.165	0.676	0.240	38.978	LPE	LPE	0.969
181	GT02-390	0.951	0.219	0.769	3.331	0.731	0.231	45.530	LPE	LPE	0.734
182	GUC10	0.948	0.224	0.761	3.222	0.724	0.239	45.807	LPE	LPE	0.986
183	GUC16	0.937	0.231	0.754	3.070	0.706	0.246	42.033	LPE	LPE	0.993
184	GUC2	0.975	0.241	0.752	3.059	0.734	0.248	39.430	LPE	LPE	0.903
185	GUC21	0.907	0.208	0.769	3.349	0.698	0.231	44.137	LPE	LPE	0.891
186	GUC3	1.018	0.240	0.763	3.226	0.778	0.237	44.770	LPE	LPE	0.622
187	GUC31	0.978	0.228	0.764	3.261	0.748	0.236	46.444	LPE	LPE	0.897
188	GUC35	0.905	0.218	0.758	3.179	0.687	0.242	45.830	LPE	LPE	0.999
189	GUC7	0.929	0.219	0.765	3.255	0.710	0.236	42.841	LPE	LPE	0.929
190	LC05-129	0.984	0.239	0.757	3.143	0.745	0.243	41.630	LPE	LPE	0.851
191	ROC16	0.831	0.194	0.767	3.308	0.637	0.233	43.622	LPE	LPE	0.997
192	Shuidian25	0.958	0.230	0.757	3.143	0.728	0.243	43.122	LPE	LPE	0.977
193	TB1	0.977	0.245	0.750	3.026	0.731	0.250	42.804	LPE	LPE	0.991
194	TB11	0.938	0.228	0.757	3.138	0.710	0.243	47.048	LPE	LPE	0.999
195	YC64-389	0.973	0.224	0.769	3.347	0.749	0.231	45.396	LPE	LPE	0.472
196	YG24	0.973	0.233	0.761	3.208	0.740	0.239	46.900	LPE	LPE	0.979
197	YR03-425	0.929	0.216	0.768	3.318	0.714	0.232	43.337	LPE	LPE	0.824
198	YR99-596	1.037	0.254	0.754	3.074	0.782	0.246	43.252	LPE	LPE	0.811
199	3203	0.955	0.216	0.775	3.453	0.740	0.225	39.363	MPE	MPE	0.888
200	12-1801	1.073	0.253	0.764	3.270	0.820	0.236	43.159	MPE	MPE	0.937
201	13-1105	0.983	0.226	0.771	3.399	0.757	0.229	41.004	MPE	MPE	0.865
202	13-4007	1.002	0.230	0.769	3.350	0.772	0.231	44.096	MPE	MPE	0.548
203	14-14325	0.987	0.230	0.766	3.302	0.756	0.234	40.426	MPE	MPE	0.844
204	14-18509	1.017	0.234	0.770	3.365	0.783	0.230	44.393	MPE	MPE	0.557
205	14-19220	1.078	0.239	0.778	3.512	0.839	0.222	42.715	MPE	MPE	0.600
206	14-20701	0.994	0.232	0.766	3.293	0.763	0.234	42.026	MPE	MPE	0.726

207	14-21107	1.058	0.250	0.763	3.240	0.806	0.237	43.152	MPE	MPE	0.872
208	14-2720	0.976	0.217	0.777	3.507	0.759	0.223	39.211	MPE	MPE	0.876
209	14-4315	1.106	0.264	0.762	3.213	0.843	0.238	46.707	MPE	MPE	0.762
210	15-1005	1.094	0.250	0.771	3.386	0.844	0.229	44.330	MPE	MPE	0.766
211	15-1106	0.970	0.231	0.762	3.252	0.739	0.238	37.959	MPE	MPE	0.826
212	15-2007	1.070	0.246	0.769	3.339	0.823	0.231	42.993	MPE	MPE	0.918
213	15-4513	0.969	0.221	0.772	3.404	0.748	0.228	41.633	MPE	MPE	0.736
214	15-5404	0.991	0.229	0.769	3.339	0.762	0.231	39.389	MPE	MPE	0.952
215	15-6204	1.016	0.235	0.769	3.334	0.781	0.231	42.981	MPE	MPE	0.795
216	15-791	1.104	0.261	0.763	3.254	0.842	0.237	44.759	MPE	MPE	0.920
217	15-794	1.059	0.234	0.778	3.524	0.824	0.222	41.196	MPE	MPE	0.775
218	16-064	1.002	0.230	0.771	3.382	0.772	0.229	40.726	MPE	MPE	0.924
219	16-065	1.014	0.234	0.769	3.343	0.780	0.231	43.919	MPE	MPE	0.653
220	16-066	0.983	0.225	0.768	3.351	0.758	0.232	42.444	MPE	MPE	0.676
221	16-0914	1.041	0.244	0.766	3.319	0.797	0.234	41.981	MPE	MPE	0.936
222	16-0928	1.081	0.251	0.767	3.356	0.830	0.233	46.285	MPE	MPE	0.578
223	16-0936	1.123	0.266	0.761	3.213	0.858	0.239	38.881	MPE	MPE	0.999
224	16-0939	1.101	0.244	0.778	3.527	0.858	0.222	41.567	MPE	MPE	0.803
225	16-0953	1.148	0.288	0.752	3.054	0.864	0.248	43.374	MPE	MPE	0.938
226	16-0954	1.069	0.252	0.765	3.266	0.817	0.235	43.230	MPE	MPE	0.927
227	16-12506	1.059	0.242	0.772	3.403	0.817	0.228	44.085	MPE	MPE	0.678
228	16-1330	1.085	0.255	0.764	3.256	0.831	0.236	44.556	MPE	MPE	0.890
229	16-136	1.070	0.254	0.763	3.235	0.816	0.237	44.667	MPE	MPE	0.809
230	16-15220	1.079	0.249	0.769	3.328	0.830	0.231	43.785	MPE	MPE	0.884
231	16-1612	1.000	0.226	0.772	3.443	0.774	0.228	42.663	MPE	MPE	0.677
232	16-167	0.981	0.231	0.767	3.307	0.752	0.233	41.663	MPE	MPE	0.718
233	16-186	1.093	0.266	0.755	3.110	0.827	0.245	42.593	MPE	MPE	0.851
234	16-187	1.035	0.240	0.765	3.309	0.795	0.235	44.833	MPE	MPE	0.620
235	16-222	1.006	0.234	0.767	3.341	0.772	0.233	44.678	MPE	MPE	0.474
236	16-223	1.05	0.247	0.765	3.279	0.803	0.235	38.485	MPE	MPE	0.993
237	16-224	1.029	0.239	0.767	3.306	0.790	0.233	45.993	MPE	MPE	0.401
238	16-22402	1.054	0.244	0.769	3.374	0.810	0.231	44.319	MPE	MPE	0.733
239	16-256	0.981	0.228	0.767	3.307	0.753	0.233	39.237	MPE	MPE	0.920
240	16-453	1.016	0.237	0.768	3.346	0.780	0.232	43.178	MPE	MPE	0.778
241	16-7019	1.115	0.260	0.767	3.310	0.854	0.233	45.452	MPE	MPE	0.839
242	16-7705	1.104	0.255	0.769	3.332	0.849	0.231	41.067	MPE	MPE	0.989
243	16-7722	0.946	0.219	0.768	3.349	0.727	0.232	40.667	MPE	MPE	0.630
244	16-832	1.004	0.224	0.777	3.503	0.780	0.223	38.430	MPE	MPE	0.948
245	16-8801	1.154	0.275	0.762	3.208	0.879	0.238	45.993	MPE	MPE	0.946
246	GT92-66	1.094	0.260	0.762	3.234	0.834	0.238	38.600	MPE	MPE	0.998
247	GT94-119	1.080	0.248	0.768	3.364	0.832	0.232	45.981	MPE	MPE	0.585
248	GUC17	0.976	0.220	0.775	3.457	0.756	0.225	40.811	MPE	MPE	0.793

249	GUC25	1.061	0.241	0.769	3.348	0.815	0.231	41.819	MPE	MPE	0.952
250	GUC29	0.988	0.224	0.774	3.429	0.765	0.226	40.126	MPE	MPE	0.910
251	GUC41	1.000	0.222	0.779	3.525	0.779	0.221	39.548	MPE	MPE	0.842
252	GUC8	0.971	0.224	0.768	3.37	0.747	0.232	43.381	MPE	MPE	0.448
253	GZ96-126	1.093	0.261	0.761	3.205	0.832	0.239	45.681	MPE	MPE	0.780
254	ROC27	1.117	0.264	0.764	3.260	0.853	0.236	46.374	MPE	MPE	0.832
255	Taiyin14	1.045	0.246	0.763	3.274	0.799	0.237	43.893	MPE	MPE	0.766
256	TB3	1.084	0.256	0.763	3.244	0.828	0.237	43.181	MPE	MPE	0.948
257	Xi096	1.043	0.24	0.769	3.358	0.803	0.231	45.048	MPE	MPE	0.581
258	ZZ9	1.040	0.240	0.768	3.323	0.800	0.232	41.470	MPE	MPE	0.952

Abbreviations: AV, mean of different photosynthetic property indicators;
**Difference between discriminant analysis and clustering analysis.
