# Supplementary figures and images for: Analysis of Photosynthetic Characteristics and Screening High Light-Efficiency Germplasm in Sugarcane
Source: Plants (Basel). 2024 Feb 22;13(5):587. doi: 10.3390/plants13050587 (PMC10935250; doi:10.3390/plants13050587)

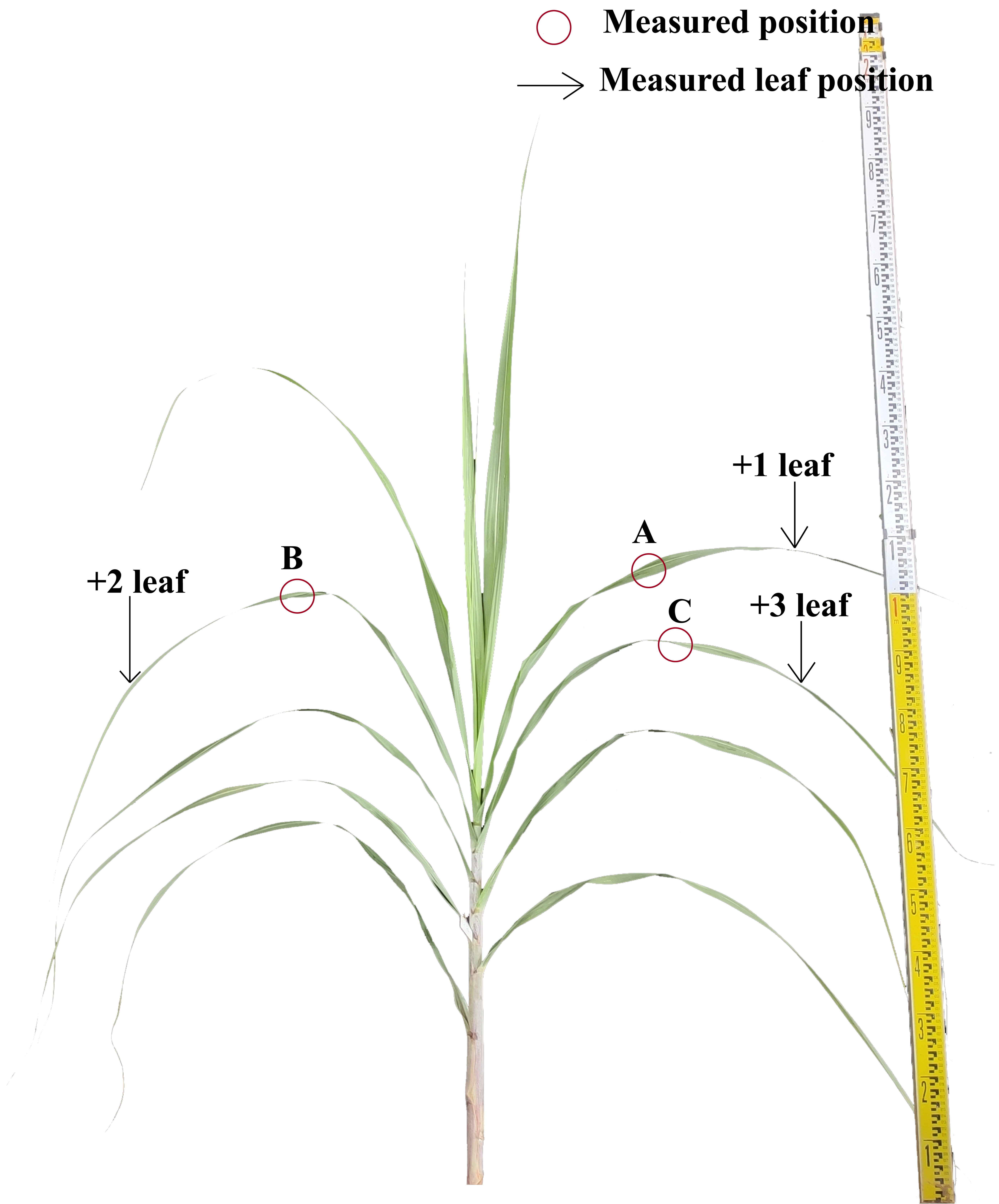

Supplement: Supplementary file 1 [file plants-13-00587-s001.zip › Figuer S1.pdf]
